# Regular Antenatal Attendance and Education Influence the Uptake of Intermittent Preventive Treatment of Malaria in Pregnancy: A Cross-Sectional Study at the University Hospital, Kumasi, Ghana

**DOI:** 10.1155/2018/5019215

**Published:** 2018-12-04

**Authors:** Otchere Addai-Mensah, Max Efui Annani-Akollor, Linda Ahenkorah Fondjo, Kwadwo Sarbeng, Enoch Odame Anto, Eddie-Williams Owiredu, Shanice Nglokie Arthur

**Affiliations:** ^1^Department of Medical Laboratory Technology, Faculty of Allied Health Sciences, College of Health Sciences, Kwame Nkrumah University of Science and Technology, Kumasi, Ghana; ^2^Department of Molecular Medicine, School of Medical Sciences, College of Health Sciences, Kwame Nkrumah University of Science and Technology, Kumasi, Ghana; ^3^University Hospital, Kwame Nkrumah University of Science and Technology, Kumasi, Ghana

## Abstract

**Background:**

The World Health Organization (WHO) recommends the use of Insecticide Treated Bed-Nets and Intermittent Preventive Treatment (IPT) with Sulphadoxine-Pyrimethamine (SP) as interventions in curbing malaria during pregnancy. However, increasing evidence shows a gap in coverage where not all pregnant women receive the recommended SP dose. This study evaluated the factors influencing uptake of IPTp-SP among pregnant women in Kumasi, Ghana.

**Methodology:**

This cross-sectional study was conducted among 280 pregnant women attending the Kwame Nkrumah University of Science and Technology Hospital in Kumasi, Ghana. Validated structured questionnaires were administered to obtain sociodemographic, medical/reproductive information, and IPTp-SP uptake among participants. Statistical analyses were performed using IBM SPSS 25.0 statistics.

**Results:**

The mean age of respondents was 29.7±4.9 years. Of the 280 women interviewed, 74.6% attended the antenatal care (ANC) clinic at least four times with only 31.8% completing the recommended doses. Tertiary education [aOR=3.15, 95% CI (0.94 -10.97), and p=0.042] and ≥ 4 ANC visits [aOR=24.6, 95% CI (5.87-103.07), p<0.0001] had statistically significant higher odds of completing the recommended IPTp-SP dose. However, participants employed by the formal sector [aOR=0.28, 95% CI (0.09 - 0.79), p=0.016] and participants with more than four children [aOR=0.14, 95% CI (0.03 - 0.63), and p=0.011] had statistically significant lower odds of completing the recommended IPT dose.

**Conclusion:**

ANC attendance is critical in IPTp uptake. The results emphasize the need for the Health Policy Makers in Kumasi to encourage pregnant women, especially women working in the formal sector and women having more than four children to patronize ANC attendance to ensure high coverage of the recommended IPTp dose.

## 1. Introduction

Pregnant women as well as the foetus are vulnerable to malaria infection [1], which has been associated with anaemia, maternal death, spontaneous abortions, low birth weight, and neonatal death [2, 3]. While malaria is a preventable and treatable disease, it continues to remain the leading cause of outpatient department (OPD) attendance in Ghana [4, 5]. It is still the most commonly reported disease, accounting for over 55.0% of all OPD attendance [5], 13.7% of admissions among pregnant women, and 3.4% of maternal deaths [6]. The first and second pregnancies are normally the most influenced. The prevalence of parasitaemia is prominent in the second and third trimesters and susceptibility to clinical malaria may persist into the postpartum period [6].

The administration of Sulphadoxine-Pyrimethamine (SP) during ANC visits as recommended by the WHO is to ameliorate the effects of malaria in pregnancy [4, 7]. ANC clinic attendance provides a planned programme for medical management of pregnant women whilst ensuring safe motherhood [8–12]. A cross-sectional study by Azizi et al. reports an association between the number of ANC visits and use of DOTs with the utilization of at least three doses of SP [13]. Another cross-sectional study involving 18,603 women aged between 15 and 49 years collected from the Malaria Indicator Surveys (MIS) conducted in Burkina Faso, Ghana, Mali, Malawi, Kenya, Nigeria, Sierra Leone, and Uganda showed an overall prevalence of 29.5% for taking three doses of IPTp-SP, with the prevalence being highest for Ghana (60%), followed by Kenya (37%) and Sierra Leone (31%) [14]. The Intermittent Preventive Treatment in pregnancy with SP is a key intervention recommended to reduce maternal malaria episodes, maternal and fetal anaemia, placental parasitaemia, low birth weight, and neonatal mortality among pregnant women living in sub-Saharan Africa [15]. IPT is provided as part of a comprehensive ANC package with other medicines like hematinics and antihelmintics. This provides maximum protection against maternal anaemia and morbidity, as well as low birth weight. It also reduces the risk of spontaneous abortion, stillbirth, and preterm deliveries [3]. The WHO recommendation where, after the first trimester, every pregnant woman should receive the recommended doses of SP at every ANC visit, beginning the first dose at 13-16th weeks of pregnancy, and the next five doses at the 20th, 26th, 30th, 34th, and 38th weeks of gestation [16], under the supervision of a health worker by Directly Observed Therapy (DOT) [15, 17].

With evidence of rise in the number of ANC visits and the adoption of the current IPTp-SP and DOT policy, it is expected that IPTp-SP uptake will be high, thereby reducing the prevalence of malaria in pregnancy and its associated adverse effects [18]. However, in Ghana, pregnant women still present with malaria and maternal anaemia [19, 20]. This study therefore evaluated the factors that influence uptake of IPTp-SP among pregnant women at the Kwame Nkrumah University of Science and Technology Hospital in Kumasi, Ghana.

## 2. Materials and Methods

### 2.1. Study Design/Setting

This was a cross-sectional study conducted at the Antenatal Care Unit of the Kwame Nkrumah University of Science and Technology (KNUST) Hospital in Kumasi, Ghana, from January to March, 2016. Kumasi is Ghana's second largest city in Ghana and lies between latitude 6.35°N and 6.40°N and longitude 1.3°W and 1.35°W. It is 150sq km in size and located in the rainforest zone of West Africa with a population of about 2 million inhabitants [21].

The Ashanti region has an intense perennial malaria transmission, with the predominant parasite being* P. falciparum*. Kumasi recorded the highest number of malaria cases (297,242 cases) in 2011 while the lowest recorded was 217,861 cases (in 2015) [22]. The prevalence of malaria in pregnancy was 36.3% in 2009 [23]. At the KNUST Hospital, the prevalence was 12.6% in 2013 [24].

### 2.2. Study Population

A total of 280 pregnant women visiting the antenatal unit of the Hospital were recruited for the study. The sample size was calculated using the Raosoft sample size calculator [25]. The minimum sample size required for this study was 196 participants at 95% confidence level, 7% margin of error, and a response distribution of 50%.

### 2.3. Ethical Considerations

Ethical approval was obtained from the Committee on Human Research Publication and Ethics (CHRPE) of the School of Medical Sciences, Kwame Nkrumah University of Science and Technology (CHPRE/RC/131/16), and the Institutional Review Board of the Kwame Nkrumah University of Science and Technology Hospital. All participants gave their written informed consent after the aim of the study had been explained to them.

### 2.4. Inclusion and Exclusion Criteria

Pregnant women of all ages who consented after the aim and objectives had been explained to them were recruited for the study. Critically ill-pregnant women were excluded.

### 2.5. Questionnaire Administration

A well-structured questionnaire was validated and administered to the study participants within the framework of the study variables consisting of demographic information (age, residence, and parity), socioeconomic status (occupation, education, and religion), the awareness of the IPT program, ANC visit, and IPTp-SP doses. Data were collected using an investigator-administered questionnaire in a language that they could easily comprehend.

### 2.6. Data Analysis

All categorical variables were presented as frequencies (percentages) and continuous variables as Means ± SD. Multivariate logistic regression analysis was used to evaluate the factors that influence ANC visit and completion of the recommended IPTp-SP dose. A p-value < 0.05 was considered statistically significant. All statistical analyses were performed using SPSS version 25.0.

## 3. Results

Table 1 shows the sociodemographic characteristics and IPTp-SP uptake of the study population

The mean age of the study population was 29.7 ± 4.9 years. The highest proportion of the participants were 26-30 years old (40.7%), had primary education (44.3%), were Christians (75.7%), employed in the informal sector (61.1%), married (85.0%), and primiparous (31.4%), and took only one IPTp-SP dose (38.6%) (Table 1).

The association between sociodemographic characteristics, medical history, and ANC visits is shown in Table 2. Cohabitation [aOR= 9.2, 95% CI (0.83-101.94)] was associated with increased odds of ANC visits and having three children [aOR = 0.23, 95% CI (0.04-1.19)] was associated with decreased odds of ANC visits, with borderline significance (Table 2).

Table 3 shows association between sociodemographic characteristics, medical history, and ANC IPT dose completion. The age of the participants, their religion and marital status, and directly observed therapy did not significantly influence the IPT dose completion. Pregnant women with tertiary education [aOR=3.15, 95% CI (0.94 -10.97), and p=0.042] and ≥ 4 ANC visits [aOR=24.6, 95% CI (5.87-103.07), and p<0.0001] had statistically significant higher odds of completing the recommended IPT dose compared to the uneducated and those informed through the clinic respectively. However, participants employed by the formal sector [aOR=0.28, 95% CI (0.09 - 0.79), and p=0.016], being primiparous [aOR=0.33, 95% CI (0.14 - 0.77), p=0.010], and women with more than four children [aOR=0.14, 95% CI (0.03 - 0.63), and p=0.011] had statistically significant lower odds of completing the recommended IPT dose (Table 3).

## 4. Discussion

ANC attendance and, subsequently, the uptake of intermittent preventive treatment to reduce the prevalence of malaria in pregnancy are expected to improve with the implementation of the current IPTp-SP and DOT policy. Nevertheless, current studies in Ghana show that the prevalence of malaria in pregnancy is still high [26, 27]. At the KNUST Hospital, the prevalence was 12.6% in 2013 [24]. This study therefore evaluated the factors that influence uptake of IPTp-SP among pregnant women at the University Hospital in Kumasi, Ghana.

Most of the respondents were within the child bearing age of 26-30 years, married, and primiparous (Table 1), consistent with an earlier study in Ghana by Doku and coworkers [28].

It was observed from this study that ANC attendance ranged from 2-12 visits with most of the participants (74.6%) making four or more visits (Table 2). This study shows an increased ANC attendance, similar to cross-sectional studies by Gross et al. [29] in Tanzania and Bouyou-Akotet et al., in Gabon [30]. This high attendance rate offers the potential for implementing the nationally recommended approaches to curbing malaria among Ghanaian pregnant women. Additionally, WHO also recommends that, after the first trimester, every pregnant woman should receive the recommended doses of SP at every ANC visit, beginning the first dose at 13-16th weeks of pregnancy and the next five doses at the 20th, 26th, 30th, 34th, and 38th weeks of gestation [16].

Findings from this study also show that cohabiting respondents [aOR= 9.2, 95% CI (0.83-101.94)] were more likely to attend the ANC compared to single women (Table 2). This corroborates a study in South-Eastern Tanzania which reported that marital status is an independent factor that influences the frequency of ANC visits [29]. Furthermore, the same study identified that not being supported by a spouse was a factor associated with a late antenatal care enrollment, buttressing the effect of companionship on ANC attendance [29]. Moreover, a cross-sectional study conducted in Ghana noted that husbands played an economic role in the frequency of a pregnant women's ANC visits with some men taking up the responsibility of maintaining the well-being of their pregnant wives by ensuring that they attend ANC clinics, whiles adolescent pregnant girls are less likely to attend an ANC and seek timely healthcare [31, 32] due to stigmatization and financial constraints.

The World Malaria Report has over the years indicated an improvement in IPT coverage among pregnant women in sub-Saharan Africa, with a recent coverage of 19% in 2016 [18]. According the Demographic Health Survey, Ghana made significant strides in improving IPT uptake from 26.8% in 2008 to 38.5% in 2014 [33, 34]. This current study observed a higher coverage of 31.8% (Table 3). The coverage in this study is lower compared to a recent cross-sectional hospital-based study by Owusu-Boateng, and Anto among Ghanaians [35]. They reported a higher coverage of IPT3 (87.5%), IPT4 (55.7%), and IPT5 (14.5%); a discrepancy that may be attributed to the fact that their study was conducted in the national capital, Accra, where most people are educated and policy implementation is effective compared to our study area. Notwithstanding the higher coverage in this, there is the need for improved IPT administration, as the coverage is far below the targeted coverage of 100% [36].

Anchang-Kimbi et al., in 2014, suggested that late first ANC clinic enrollment and fewer clinic visits may prevent the uptake of SP; however education on early and regular ANC clinic visits may increase IPTp coverage [37]. Other studies have however reported a negative influence of education on ANC visit and IPTp –SP uptake [38, 39]. This study, on the contrary, observed that pregnant women with tertiary level of education [aOR=3.15, 95% CI (0.94 -10.97), and p=0.042] were more likely to complete the recommended IPTp-SP dose in comparison to the uneducated (Table 3), consistent with other studies [40–45]. This association may be attributed to the ability of the educated to understand health information as well as its significance and hence adherence to the recommended dose [46–48].

However, women who worked in the formal sector [aOR=0.28, 95% CI (0.09 - 0.79), and p=0.016] were less likely to complete recommended SP dose compared to the informally employed (Table 3). This may be attributed to the fact that women employed in the formal sector work under strict conditions and are not at liberty to leave their jobs to attend the antenatal clinics [29].

Our study also shows that primiparous women were less likely to complete the recommended dose of SP [aOR=0.33, 95% CI (0.14 - 0.77), and p=0.010] compared with women with no child (Table 3). This finding is in consonance with reports by Kuile et al, [49] and Olorunda, [50] who indicated that have only one child are less likely to use malaria preventive measures due to their younger age. Moreover, women with more than four children [aOR=0.14, 95% CI (0.03 - 0.63), p=0.011] were also less likely to complete the recommended IPTp-SP dose (Table 3). Furthermore, higher number of ANC visits [aOR=24.6, 95% CI (5.87-103.07), p<0.0001] positively influenced the uptake of the recommended IPT dose (Table 3). This is in consonance with a cross-sectional study by Bouyou-Akotet et al., in 2013 among Gabonese [30], and Nkoka et al. among Malawian women [51]. Nonetheless, the finding of this study contradicts a cross-sectional study by Toure et al. in Côte d'Ivoire [52]; however, SP stock shortage, absence of cups and clean drinking water for taking SP by DOT, unclear guidelines or confusion among health workers regarding the guidelines, and other related health facility factors were the factors attributed to the hindrance of the IPTp delivery.

This study is limited by the fact that some of the responses provided by the participants may not be accurate due to fear of incrimination. However, participants were assured of privacy and confidentiality of the information they provided. Another limitation of this study is that it was done in an urban setting and might not be generalizable to other areas especially rural areas. Furthermore, factors such as availability of drugs at the health facility and health workers' performance that may have influence IPT-SP uptake were not assessed. Moreover, the study area was a large tertiary hospital and women might be from different areas that might differ in malaria endemicity.

## 5. Conclusion

ANC attendance is critical in IPTp uptake. The results emphasize the need for the Health Policy Makers in Kumasi to encourage pregnant women, especially women working in the formal sector and women having more than four children to patronize ANC attendance to ensure high coverage of the recommended IPTp dose.

## Figures and Tables

**Table 1:** Sociodemographic characteristics and IPTp-SP uptake of the study population.

Variable	Frequency (n=280)	Percentage (%)
**Age (Years)**	29.71± 4.9	
15-20	4	1.4
21-25	47	16.8
26-30	114	40.7
31-35	77	27.5
36-40	34	12.1
41-45	4	1.4
**Educational Status**		
No education	20	7.1
Primary	124	44.3
Secondary	77	27.5
Tertiary	59	21.0
**Religion**		
Christian	212	75.7
Muslim	68	24.3
**Occupation**		
Unemployed	38	13.6
Informal	171	61.1
Formal	69	24.6
**Marital Status**		
Single	18	6.4
Married	238	85.0
Cohabiting	24	8.6
**Parity**		
0	62	22.1
1	88	31.4
2	72	25.0
3	30	10.7
≥4	28	10.0
**IPTp-SPdoses**		
0	8	2.9
1	108	38.6
2	75	26.8
3	89	31.8

IPTp-SP; intermittent preventive treatment in pregnancy with sulphadoxine-pyrimethamine.

**Table 2:** Association between sociodemographic characteristics, medical history, and ANC visits.

Variable	≤ 3 visits; 71(25.4%)	≥ 4 visits; 209(74.6%)	aOR (95% CI)	p-value
**Age(years)**				
15-20	0(0)	4(100.0)	1 (reference)	
21-25	12(25.5)	35(74.5)	3.02(0.14-63.91)	0.477
26-30	31(27.2)	83(72.8)	2.36(0.13-41.96)	0.557
31-35	17(22.1)	60(77.9)	4.49(0.25-79.03)	0.304
36-40	9(26.5)	25(73.5)	4.05(0.22-73.76)	0.345
41-45	2(50.0)	2(50.0)	1.00(0.06-15.98)	1.000
**Education**				
No education	5(25.0)	15(75.0)	1 (reference)	
Primary	36(29.0)	88(70.1)	1.42(0.33-6.06)	0.639
Secondary	16(20.8)	61(79.2)	1.61(0.33-7.89)	0.559
Tertiary	14(23.7)	45(76.3)	1.04(0.14-7.55)	0.971
**Religion**				
Christian	55(25.9)	157(74.1)	1 (reference)	
Muslim	16(23.5)	52(76.5)	2.07(0.86-5.00)	0.106
**Occupation**				
Unemployed	4(10.5)	34(89.5)	1 (reference)	
Informal	50(28.6)	125(71.4)	0.62(0.19-2.31)	0.518
Formal	17(25.0)	51(75.0)	0.57(0.12-2.63)	0.481
**Marital Status**				
Single	4(22.2)	14(77.8)	1 (reference)	
Married	66(27.7)	172(72.3)	2.12(0.38-11.69)	0.389
Cohabiting	1(4.2)	23(95.8)	9.2(0.83-101.94)	0.07
**Parity**				
0	11(17.7)	51(82.3)	1 (reference)	
1	21(23.9)	67(76.1)	0.88(0.27-2.97)	0.849
2	18(25.0)	54(75.0)	0.55(0.13-2.21)	0.402
3	9(30.0)	21(70.0)	0.23(0.04-1.19)	0.079
>4	12(42.9)	16(57.1)	0.42(0.080-2.21)	0.307
**Supervision(DOT)**				
Yes	66(24.8)	200(75.2)	1 (reference)	
No	5(35.7)	9(64.3)	1.87(0.59-5.89)	0.288

DOT: directly observed therapy. Chi square test was performed to compare categorical variables. Multivariate logistic regression analysis was used to evaluate the effect of sociodemographic characteristics and medical history on ANC visits. *p* < 0.05 was considered statistically significant (*p* values of significant variables are in bold print).

**Table 3:** Association between sociodemographic characteristics, medical history, and ANC IPT dose completion.

Variables	Complete; 89(31.8%)	Incomplete; 191(68.2%)	aOR (95% CI)	p- value
**Age**				
15-20	3(75.0)	1(25.0)	1 (reference)	
21-25	14(29.8)	33(70.2)	0.10 (0.01 - 1.61)	0.105
26-30	35(30.7)	79(69.3)	0.11 (0.01 -1.98)	0.138
31-35	25(32.5)	52(67.5)	0.15 (0.01 - 2.73)	0.202
36-40	11(32.4)	23(67.6)	0.22 (0.01 - 4.09)	0.308
41-45	1(25.0)	3(75.0)	0.23 (0.01 - 10.63)	0.457
**Education**				
Uneducated	4(20.0)	16(80.0)	1 (reference)	
Primary	30(24.2)	94(75.8)	1.28 (0.40 – 4.12)	0.682
Secondary	29(37.7)	48(62.3)	2.42 (0.74 – 7.94)	0.188
Tertiary	26(44.1)	33(55.9)	3.15 (0.94 -10.97)	**0.042**
**Religion**				
Christian	69(32.5)	143(67.5)	1 (reference)	
Muslim	20(29.4)	48(70.6)	0.97 (0.48 - 1.98)	0.954
**Occupation**				
Unemployed	18(47.4)	20(52.6)	1 (reference)	
Informal	48(27.0)	130(73.0)	0.72 (0.31 - 1.73)	0.469
Formal	23(35.9)	41(64.1)	0.28 (0.09 - 0.79)	**0.016**
**Marital status**				
Single	7(38.9)	11(61.1)	1 (reference)	
Married	74(31.1)	164(68.9)	1.69 (0.49 - 5.76)	0.400
Co-habiting	8(33.3)	16(66.7)	1.38 (0.29 - 6.53)	0.681
**Parity**				
0	30(48.4)	32(51.6)	1 (reference)	
1	22(25.0)	66(75.0)	0.33 (0.14 - 0.77)	**0.010**
2	25(34.7)	47(65.3)	0.52 (0.20 - 1.33)	0.174
3	8(26.7)	22(73.3)	0.34 (0.10 - 1.14)	0.081
≥ 4	4(14.3)	24(85.7)	0.14 (0.03 - 0.63)	**0.011**
**Number of ANC visits**				
≤ 3	2(2.8)	69(97.2)	1 (reference)	
≥ 4	87(41.6)	122(58.4)	24.6(5.87-103.07)	**<0.0001**
**Supervision (DOT)**				
Yes	86(32.3)	180(67.7)	(reference)	
No	3(21.4)	11(78.6)	1.81 (0.42 - 7.81)	0.425

Complete: completion of recommended dose, ANC: antenatal care, and DOT: directly observed therapy. Multivariate logistic regression analysis was used to evaluate the effect of sociodemographic characteristics and medical history on ANC IPTp-SP dose completion. *p* < 0.05 was considered statistically significant (*p* values of significant variables are in bold print).

## Data Availability

All relevant data are within the article.
